# Psychometric Properties and Measurement Invariance for a Chinese Version of a Psychological Need Thwarting Scale for Teachers

**DOI:** 10.3390/ijerph17093247

**Published:** 2020-05-06

**Authors:** Liang Chen, Jeffrey Hugh Gamble, I-Hua Chen, Zeng-Han Lee, Qian-Lan Fu

**Affiliations:** 1 School of Education, Shaanxi Normal University, Xi’an City 710062, China; chenliang_0801@126.com; 2 Department of Foreign Languages, National Chiayi University, Chiayi 62103, Taiwan; 3 College of Education Science, Minnan Normal University, Zhangzhou 363000, China; 4 College of Teacher Education, Wenzhou University, Wenzhou City 325035, China; zenghan88@gmail.com; 5 Qionglai Nanjie Primary School, Qionglai City 611500, China; lanlan08070317@sina.com

**Keywords:** psychological need thwarting, burnout, emotional exhaustion, psychometrics, Chinese basic education, measurement invariance

## Abstract

While teachers’ psychological needs have been evaluated in terms of need satisfaction, need thwarting of teachers is under-researched. This study developed a Chinese version of a Psychological Need Thwarting (PNT) scale for teachers and evaluated both its psychometric properties and measurement invariance across groups. Psychometric criteria for the scale were evaluated, with satisfactory levels of internal reliability, test–retest reliability, convergent and divergent validities, and model goodness-of-fit. One item translated from the original PNT scale was removed due to cross-loading. Criterion validity was established, with *R^2^* = 0.54 for the factor of burnout (emotional exhaustion). Measurement invariance was established using confirmatory factor analysis for the factors of gender, grade of instruction, and position. The teachers evaluated demonstrated higher levels of competence thwarting, as compared to autonomy and relatedness thwarting, but overall higher levels of thwarting as compared to previous research. Males reported higher levels of autonomy and competence thwarting as compared to females and secondary school teachers reported higher levels of relatedness thwarting as compared to primary school teachers. The developed scale can serve as a valuable tool in evaluating the thwarting of teachers’ psychological needs, an issue which can profoundly impact teachers’ and students’ mental health and performance.

## 1. Introduction

### 1.1. Background

Teachers’ enthusiasm for their profession is a prerequisite for successful teaching. In the Teaching and Learning International Survey (TALIS) held by the Organisation for Economic Co-operation and Development for 2013 and 2018, teachers’ identification as “professionals,” involving aspects such as leadership, networking, collegial interaction, and autonomy, was emphasized as a fundamental factor in terms of teachers’ job satisfaction and intention to continue teaching [1,2]. On the contrary, for teaching professionals, feelings resulting from “burnout” not only cause harm to teachers’ personal quality of life but also have negative impacts on students [1]. Previous studies have shown that teacher burnout is significantly associated with depression and turnover intention [3,4], negatively impacting students’ learning motivation [5]. Teachers’ stress can even negatively influence students’ own physiological regulation of stress, with observed increases in students’ levels of cortisol during class serving as a biological symptom of stress [6].

Given the negative impact of burnout on both teachers and students, it is necessary to investigate the determinants of teachers’ job-related burnout. Teacher burnout is characterized by long-term stress in three areas: exhaustion caused by a lack of support in dealing with emotion, a sense of depersonalization and detachment towards one’s work, and a sense of professional inadequacy [7]. As such, scholars believe that burnout is intimately related to the school environment, with Skaalvik and Skaalvik [8] finding that schools’ overemphasis on performance goals increase teachers’ workload, resulting in the emotional exhaustion associated with job burnout. In a meta-analysis of teacher burnout by Iancu et al. [9], the role of school administration in teacher burnout was also emphasized, particularly concerning the negative impact of lack of resources and support and insufficient opportunities for collaboration and communication with colleagues. In less developed countries, on the other hand, one potential predictor of teacher burnout was a lack of teacher autonomy [9].

From the perspective of self-determination theory (SDT), Ryan and Deci [10] describe three environmental supports required to meet teachers’ fundamental psychological needs: competency, autonomy, and relatedness [10]. As such, when teachers lack autonomy in their classroom or school,(i.e., they are burdened with expectations, which exceed their personal competence due to excessive performance requirements) and are offered insufficient social support (relatedness), an overall negative perception towards the school and one’s own profession is formed. This negative perception results largely from the thwarting of teachers’ basic psychological needs: autonomy, competence, and relatedness. As such, when the environment fails to meet teachers’ psychological needs or even actively frustrates these needs, teachers can easily fail to adapt, resulting in negative outcomes (i.e., burnout). SDT, while emphasizing the social and contextual influences on psychological needs, recognizes the potential of human beings as proactive, motivated, and self-regulated beings who, despite the absence of psychological need satisfaction or the presence of psychological need thwarting, through personal resilience and self-management can overcome these challenges to varying degrees. Nevertheless, when considering the high levels of teacher burnout observed in many countries, including China, evaluating the psychological needs of autonomy, competence, and relatedness is an area that requires much deeper investigation.

In most earlier studies in the area of SDT, scholars paid more attention to the positive effects brought about by the environment in terms of teachers’ psychological need satisfaction (PNS), and found that factors including work engagement [11], positive attitude towards school life [12], and overall well-being [13] were all significantly related to PNS. In contrast, there has been much less discussion on psychological need thwarting (PNT), characterized by disempowerment, greater levels of coercion and pressure, and lack of social support [14]. Research evaluating job demands and job resources found that the influence of job demands (including work load and time pressure) were negative, concerning teacher well-being, and outweighed the potential benefits of job resources [15]. The role of personal resources (including self-efficacy and coping resources) in addition to job resources (including autonomy, support from colleagues, and feedback) was related to both positive outcomes, including engagement, as well as negative outcomes, including burnout [16]. Thus, personal and job resources, in addition to systemic variables, must be considered in the context of teacher burnout resulting from psychological need thwarting.

From among the few studies evaluating PNT, most have focused on students taking physical education courses or athlete training [17,18,19,20,21] with fewer studies evaluating teachers’ PNT within the larger community of learning. As a result, oversimplistic approaches to defining and conceptualizing PNT may result in the view that PNT can be viewed simply as the equivalent to low PNS, without the need for the development of an independent construct, PNT. The work of Vansteenkiste and Ryan [22] has demonstrated how, within the framework of SDT, PNS is related to aspects of well-being and individual growth, while PNT is more related to ill-being, attempts to fulfil alternative needs, or general inability to function well. Furthermore, Costa, Ntoumanis, and Bartholomew [23] directly state that “a lack of need satisfaction (i.e., need dissatisfaction) is not equivalent to experiences of need thwarting.” In response, recent SDT research has clarified the distinction between PNS and PNT as two relatively independent constructs, as in the research of Gunnell et al. [19] who found that while PNS does have significant explanatory power in predicting positive emotional well-being and feelings of energy, PNT demonstrated a stronger effect than PNS. Furthermore, the correlation between PNS and PNT was found to have only a small to moderate effect (i.e., correlation coefficients from −0.10 to −0.54), indicating that PNS and PNT do not belong on the same dimension [19]. From the research of Sheldon, Abad, and Hinsch [24], satisfaction and dissatisfaction in terms of “relatedness” were both significantly and positively associated with duration of social media use, suggesting that it is not possible for PNT to simply be considered as the lack of PNS. Since both variables, PNS and PNT, were significantly and positively correlated with the same outcome (Facebook use time), it is evident that a lower degree of PNS is not equivalent to a higher degree of PNT and vice versa.

Recently, scholars have begun to systematically examine the negative effects of PNT (e.g., sense of ill being, higher levels of stress, and fatigue) [25,26,27]. In order to accurately evaluate the construct of PNT, a valid and reliable measurement scale is vital. Looking back on prior studies on PNT scale development, Bartholomew et al. were the first researchers to develop the “Psychological Need Thwarting Scale” (PNTS) [20], which was used to measure the psychological need frustration experience of athletes during training. The PNTS was developed in accordance with the original three-factor SDT framework proposed by Ryan and Deci [10], and resulted in indices for goodness-of-fit, internal consistency, and reliability that meet psychometric standards for scale development [20]. In order to make the scale applicable to teachers, Bartholomew et al. [27] made further minor modifications to the wording of the scale and investigated the teacher version of the scale by establishing a structural equation model of PNT, including the factors of work pressure and job burnout. Although Bartholomew et al. mentioned that the teacher version of the PNTS met appropriate psychometric standards [27], their specific evaluation criteria were not described in sufficient detail. Thus, the only research that currently contains a complete review of the PNTS’s psychometric testing criteria, as applied to teachers’ PNT, is a study by Cuevas et al. [28], which examined the reliability and validity of the PNTS among Spanish physical education teachers.

According to Bartholomew et al. [20], appropriate measurement tools for psychological needs thwarting are still lacking in the literature on SDT, mentioning that PNT is context specific and, therefore, PNT scales should be developed for specific situations and contexts. The fact that empirical studies of PNT based on the original PNTS [20] have either a) required modifications to the scale after empirical examination or b) resulted in findings which were not consistent with the original PNTS study may be due to this need for contextualization. As an example, in a study evaluating the French version of the PNTS, as translated by Martinent et al. [17], one-factor loading belonging to the “relatedness” factor of need thwarting was too low (i.e., 0.27), a finding that was not reported by the original study by Bartholomew et al. [20]. Thus, in order to improve the model fit, Martinent et al. [17] chose to delete that item. Likewise, when Liu et al. [18] developed a PNT scale for middle school physical education students in China, one item from the original PNTS was directly deleted in order to achieve goodness-of-fit. 

Based on the few available studies evaluating PNT among teachers, it is clear that the development of teachers’ PNT measurement tools must be carried out with respect to context, including cultural and national differences; as such, it is not feasible or appropriate to directly adopt the original content of the PNTS designed by Bartholomew et al. [20]. Furthermore, not only must PNT measurement tools be context specific, in regards to the application of SDT to research and practice, but social and cultural factors must also be considered. Specifically, the influence of cultural differences between Eastern and Western educational systems on the theoretical relevance and applicability of SDT have remained a focus of scholars’ attention [29,30]. One fundamental difference impacting the interpretation and measurement of SDT-related factors is the emphasis of traditional Chinese culture on social harmony over the autonomy and independence which is valued more highly by Western cultures, generally speaking [31]. In addition, due to the influence of culture on pedagogy, differences between Eastern teachers and Western teachers have been shown to exist for career motivation and degree of self-determination [29]. Therefore, although, in terms of PNS, the fulfillment of teachers’ needs for autonomy can lead to positive results for Chinese teachers [11], such as work engagement, the degree to which the results from studies on teachers’ PNT can apply cross-culturally is unclear. Therefore, in terms of the evaluation of teachers’ PNT in the Chinese context, further examination is required in order to develop a PNT scale with good reliability and validity for use with teachers in China.

### 1.2. Research Purpose

Teachers in China’s basic education system currently account for the largest teaching population in the world. According to statistics from the World Bank, in 2018, there were 620,151 primary school teachers in China [32]. Despite such a large population of teachers, no suitable Chinese version of a PNT scale for teachers has been developed yet. Furthermore, due to educational reforms and changes in the social climate, such as the levels of teacher turnover observed in recent years, the Chinese government launched a policy in 2018 entitled “Opinions on Deepening the Reform of Teaching Staff in the New Era,” which aims to “attract outstanding talents to become teachers” [33]. Thus, this current situation of high turnover in China’s basic education provides an optimal chance to explore the PNT of China’s teachers, as instabilities in teachers and their communities of learning suggest that the school system is largely failing to meet teachers’ psychological needs. In addition, a deeper evaluation of the PNT of Chinese teachers can further extend an ongoing discussion in the literature regarding issues of cross-cultural generalizability from SDT studies [34]. Noteworthy issues for investigation include whether or not the construct of PNT among Chinese teachers is composed of the same three independent factors regarding the thwarting of psychological needs as Bartholomew et al. [20] and the degree to which the PNT of Chinese teacher can predict burnout as compared to the work of Cuevas et al. [28]. Thus, the purpose of this study is to evaluate and verify these important issues. 

In order to address the above issues, the first step of this study was to develop a reliable and valid tool for measuring the PNT of teachers in China. As such, one main purpose of this study was to develop and validate a Chinese version of a PNT scale for teachers (Chinese Psychological Need Thwarting of Teachers; CPNTT) which could be used to evaluate PNT among Chinese basic education teachers. Considering that the PNTS designed by Bartholomew et al. [20] was based on the SDT framework, had a solid theoretical foundation, and obtained good reliability and validity when evaluated in other Western countries, the items from the PNTS were adopted as a model for the development of a Chinese version. In addition to testing the construct validity of the scale developed in this study, burnout served as the criterion variable for testing the criterion validity of the CPNTT. Finally, multiple groups measurement invariance was conducted using the background demographics of teachers, specifically in terms of gender, grade of instruction (elementary, junior, or high school), and position (with or without concurrent administrative duties). These factors were selected due to their potential applicability for future studies comparing the means of different groups based on data from other populations.

## 2. Materials and Methods

### 2.1. Participants

This study was approved by the research ethics committee of a local university in Shandon province (IRB No. BG18-0320). According to the purposes of this study, participants were divided into two groups. The first group was utilized for a pilot test, serving as participants for a 6-month follow-up evaluation. A public elementary school in a county of Sichuan province in China was selected for convenience sampling of participants for this first group (Group 1), which consisted of 141 teachers who agreed to participate in the survey to examine the preliminary psychometric characteristics of the CPNTT in order to calculate test–retest reliability. Among these teachers, 124 were female and 17 were male. The average age was 36.97(*SD* = 10.04), and the average years of teaching experience was 16.09 (*SD* = 12.31).

The second group was a formal sample obtained through cluster sampling of all 14 provinces in China. An online questionnaire was completed using smart phones. The reason for conducting an online survey was due to the need to protect the privacy of the participants, which was particularly important given the sensitive nature of the items included in the CPNTT that included questions designed to measure negative perceptions toward schools. A total of 1535 in-service teachers participated in this study. There were no missing data for the responses from each participant, due to the function of the online survey platform requiring participants to fill out each question before final submission. The formal participants (Group 2) were from elementary schools (1095), junior high schools (314), and senior high schools (126). In particular, 60.5% (928) of the participants were female, and most of the participants came from public schools (98.1%). The average age of respondents was 40.84 (*SD* = 9.78), and the average years of teaching experience was 19.16 (*SD* = 11.13). Teachers mainly taught Chinese (30.42%), Mathematics (40%), and English (6.91%) as their primary subjects. The proportion of teachers of music, sports, art, information technology, science, and other subjects was between 2% and 5%. Overall, 18.4% of teachers had concurrent administrative positions. Finally, due to the use of cluster sampling in this study, in order to avoid the possible influence of nested data, the intraclass correlation coefficient (ICC) of the three factors of the CPNTT was calculated among teachers from different provinces. The ICC values of the three factors were 0.03, 0.003, and 0.05, less than the value of 0.059 [35], which suggests no interference of the nested data on the analysis.

### 2.2. Measures

The CPNTT was translated from Bartholomew et al.’s PNTS [27] for teachers, with the Spanish version of the PNTS by Cuevas et al. also used as a reference [28]. The PNTS contains three subscales: “autonomy thwarting,” “competence thwarting,” and “relatedness thwarting.” Each subscale includes four items. Likert-type responses were used, ranging from 1 (strongly disagree) to 7 (strongly agree). Thus, the higher the score, the higher the degree to which a teacher’s psychological needs were thwarted. Since Bartholomew et al. [27] did not report the psychometric results for the teacher’s version of the PNTS, the results from Cuevas et al. [28], using responses from 619 middle school physical education teachers in Spain, were used as a reference. Cuevas et al. [28] noted values for RMSEA, SRMR, and CFI for the overall measurement model of 0.08, 0.05, and 0.95, respectively, which was significantly related to the criterion variables (i.e., job burnout). 

The Chinese version of the “Primary and Secondary School Teachers’ Job Burnout Questionnaire” by Wu et al. [36] was used in this study to assess the job burnout of participants. The subscale for “Emotional Exhaustion” was utilized, since this factor is the most salient in terms of job burnout [36]. In Wu et al.’s study, 1380 primary and secondary school teachers from six provinces in China were selected as subjects, and the overall measurement model demonstrated a good fit: RMSEA = 0.06, NFI = 0.95, and CFI = 0.96. Eight items were included for the subscale “Emotional Exhaustion” (e.g., “I feel like I’m overdrawn” and “I feel that my teaching work has exhausted my emotions”). The responses were reported as Likert-type responses ranging from 1 (strongly disagree) to 7 (strongly agree). The factor loading coefficients were high, from 0.69 to 0.85, and the internal consistency reliability was 0.90, demonstrating high reliability and validity.

### 2.3. Procedure for the Translation, Pilot Test, Formal Test, and Follow-Up Test

The translation of the CPNTT included the following stages. First, a back translation was utilized in order to translate the PNTS [27,28] into a Chinese version. To ensure the quality of this translation, a Canadian educator, fluent in Chinese, English, and Spanish, participated by forming the first draft of the CPNTT. Second, to ensure the validity of the questions, five experienced primary school teachers and principals were recruited to polish and revise the wording of the survey in order to make the content of the CPNTT more appropriate and comprehensible regarding the actual teaching context and circumstances of teachers in China. 

Concerning cross-cultural issues, although the back translation ensures that the meaning of each item does not deviate from the meaning implied by the original scale, the Chinese version of the item may not match the actual context of China’s education environment. Therefore, after completion of the CPNTT, a pilot test was used to evaluate the appropriateness of the items. According to the rule of thumb that the ratio of items to participants should reach 1:10, the first group of 141 primary school teachers was utilized for the pilot test. The test results demonstrated sufficient internal consistency reliability for the CPNTT, with an overall Cronbach’s α of 0.85, and Cronbach α values for the need thwarting subscales of autonomy, competence, and relatedness of 0.70, 0.80, and 0.86, respectively. Furthermore, exploratory factor analysis (EFA) computed Eigenvalues for the three factors greater than 1, with a total explained variance of 59.50%. Thus, the factor structure of the pilot test of the CPNTT demonstrated consistency with the original English version of the PNTS. 

Next, the factor of each factor loading was further examined for each item. During this procedure, it was noted that one item “due to the lack of training opportunities in the environment, I feel that I am not competent in my daily work tasks,” which was an item belonging to the “competence thwarting” subscale, had a high degree of cross-factor loading. In fact, this item was originally “I feel inadequate because I am not given opportunities to fulfill my potential” in the PNTS, but when examining the draft version of the CPNTT, the five expert local principals and teachers considered that the meaning of the item was too abstract and might prove difficult for teachers to understand, resulting in a modification of the wording. Even though the research team (including Chinese principals and teachers) believed that the revised item was clearer and did not deviate from the spirit of the PNTS, this item as deemed unsatisfactory based on the results of EFA. After discussion with the team of five principals and primary schools’ teachers, based on an understanding of the Chinese education environment, whether or not teachers have opportunities for growth and additional training depends both on the overall system (competence) and, to a certain degree, human relationships (relatedness). Therefore, the interpretation of this item involved a combination of both system-level and interpersonal-level factors within the school environment, which are not only explained by “competence thwarting” in the original PNTS but also by “relatedness thwarting.” However, considering that the CPNTT is a translated scale, the structure and content of the original scale should be maintained as much as possible. Thus, since it was deemed inappropriate to delete the item directly, it was retained, with subsequent results scrutinized more carefully.

After the completion of the pilot test, formal testing was conducted, along with follow-up surveys. The formal test was administered in the form of an online survey with the assistance of the local education bureau using a website to inform teachers and distribute the online questionnaire to the online teacher community of participating schools. A follow-up survey was administered to the Group 1 elementary teacher participants, who participated in the pilot test, after a period of 6 months. This follow-up survey was conducted in order to establish the test–retest reliability of the CPNTT.

### 2.4. Data Analysis

Descriptive statistics reported the mean and standard deviation for each subscale of the CPNTT as well as the internal reliability of each construct (Table 1). Furthermore, repeated measures ANOVA was used to compare the variance among different subscales of the CPNTT, with one sample *t*-tests used to examine whether or not significant differences were found between the responses from teachers and the calculated value of 4.45, which indicates the value above which suggests “agreement” based on a 7-point Likert-type scale, as suggested by Narli [37]. A between-subjects ANOVA was also computed to compare differences among responses to the three PNT subscales by teachers from different backgrounds, in terms of gender, grade of instruction (elementary, junior, or high school), position (with or without concurrent administrative duties), and subject taught.

Next, LISREL 8.80 was used to conduct confirmatory factor analysis (CFA) in order to validate the CPNTT. Since the previous EFA results, based on the pilot test, indicated that the factor loading of item 8 of the CPNTT was not as expected, based on the structure of the PNTS, a comparison of the fit of the CPNTT model with and without item 8 was conducted. Next, differences in the model fit indices between a single-factor model (combining the three PNT subscales) and the three-factor model were evaluated in order to determine whether the CPNTT factor structure matched the original PNTS factor structure. After selecting the best fitting factor structure, construct validity was measured in terms of both convergent and discriminant validities. In addition to reviewing the fit indices of the model, the composition reliability (CR) and average variance extracted (AVE) were computed. According to Fornell and Larcker [38] convergent validity is supported if the CR and AVE of each construct are higher than 0.50 and 0.70, respectively. Furthermore, to fully satisfy the requirements of discriminatory validity, the AVE value should be larger than the *R*^2^ (the squared correlation between two constructs). In terms of criterion-related validity, a structural equation model was established with four latent variables: the three subscales of the CPNTT and burnout. Correlations among these four latent variables were also tested.

In term of test–retest reliability, several further analyses were conducted. Values from paired *t* tests, intraclass correlation analysis (ICC, two-way random), standard error of measurement (SEM) (i.e.,(standard deviation of all test–retest scores) ×$\sqrt{1-ICC}$), smallest real difference (SRD) (i.e., 1.96 × SEM × $\sqrt{2}$), and SRD% were used to evaluate the test–retest reliability of the CPNTT. SRD% was calculated by dividing the SRD by the mean of all measurements and multiplying by 100%. In order to evaluate overall measurement error, a cutoff value of ICC was set as greater than 0.60 [39], whereas an SRD% of less than 30% [40] was deemed to indicate acceptable measurement error for items in this study. 

Finally, multiple group measurement invariance was performed based on teachers’ background variables, in terms of gender, grade of instruction (elementary, junior, or high school), and position (with or without concurrent administrative duties). Four nested models were constructed and compared to determine whether measurement invariance was supported across different groups. These models included: (1) a configural invariance model (baseline model), (2) a model with factor loadings constrained as equal (metric invariance model), (3) a model with factor loadings and item thresholds constrained as equal (scalar invariance model), and (4) a model with factor loadings, item thresholds, and construct covariance constrained as equal. Configural equivalence acts as a baseline and is supported if the factor pattern is invariant across groups. On the premise that the configural model was satisfactory, a metric invariance model was evaluated, with the constraint of equal factor loading coefficients across two groups. Following evaluation of metric invariance, scalar invariance was further tested by adding the constraint of the intercepts of all items across two groups being equal. Under scalar invariance, the mean of a construct is the same between the different groups being compared. As for the constraint of the covariances of constructs being held equal across two groups, although this is excessively rigorous [41], this procedure was adopted, considering that PNT research is still in development. As such, since scholars may want to use CPNTT to test the correlations among the three psychological need thwarting subscales in the future, measurement invariance for construct covariance was also included in this analysis.

For the CFA evaluated in this study, the model fit indices included a non-normed fit index (NNFI), comparative fit index (CFI), a root mean square error of approximation (RMSEA), and standardized root mean square residual (SRMR). According to Hu and Bentler [42], the following rigorous cutoffs were adopted: NNFI and CFI ≥ 0.90, SRMR ≤ 0.08, and RMSEA ≤ 0.10. Regarding measurement invariance, the findings are supported if the comparison between the previous and consecutive models demonstrate the following changes in indices: ΔCFI > −0.01, ΔRMSEA < 0.02, and ΔSRMR < 0.03 (for invariant loadings) or < 0.01 (for invariant intercepts and construct covariance) [43,44].

## 3. Results

### 3.1. Descriptive Statistics, Internal Reliability, t-test, and ANOVA Results

First of all, the mean of the three PNT subscales for Chinese teachers (see Table 1) from high to low were: competence thwarting (*M* = 4.24, *SD* = 1.54), autonomy thwarting (*M* = 3.91, *SD* = 1.61), and relatedness thwarting (*M* = 2.09, *SD* = 1.24). A repeated measures ANOVA showed a significant difference among the three subscales (*F*(2, 3068) = 1656.09, *p* < 0.01, and *ηp^2^* = 0.52). Post hoc results indicated that competence thwarting > autonomy thwarting > relatedness thwarting. More specifically, the results of one sample *t*-tests show that the degree of autonomy thwarting and relatedness thwarting for Chinese teachers is significantly lower than the representative value indicating agreement that their psychological needs had been thwarted (i.e., 4.45). In fact, both autonomy thwarting (*t*(1534) = −12.97, *p* < 0.01) and relatedness thwarting (*t*(1534) = −74.31, *p* < 0.01) were significantly lower than the value of 4.45 established as the threshold of agreement. However, with a mean of 4.82, competence thwarting (*t*(1534) = 8.11, *p* < 0.01) was significantly higher than the threshold for agreement.

Second, regarding differences in terms of PNT among teachers of different backgrounds, the result of between-subjects ANOVA demonstrated that differences only existed for gender and grade of instruction (see Table 1). Males reported significant higher levels of autonomy thwarting (*F*(1,1533) = 59.10, *p* < 0.01) and competence thwarting (*F*(1,1533) = 5.59, *p* = 0.02) as compared to female teachers. Junior and senior high school teachers also reported higher degrees of relatedness thwarting than primary school teachers (*F*(2,1532) = 9.81, *p* < 0.01), with no differences found between junior and senior high school teachers. 

Third, the internal reliability of each construct was evaluated (see Table 1). The Cronbach’s α for autonomy, competence, and relatedness thwarting were 0.76, 0.76, and 0.83, respectively, indicating acceptable reliability. Moreover, given the concerns in the pilot test regarding item 8, the change in reliability and mean for the dimension of competence thwarting was evaluated when this item was included or removed. The results indicate that the Cronbach’s α coefficient improved, and that the mean increased, when item 8 was removed, indicating that this item seemed to be evaluating a different construct from the other items in this factor.

### 3.2. Construct and Criterion Validity

Based on the findings noted in previous sections (i.e., the pilot test and internal reliability analysis), inclusion of item 8 may adversely affect the overall measurement model. Therefore, this study first compared the fit indices of two models (retaining vs. deleting item 8). Table 2 shows the results. First, it was found that if item 8 was retained, the overall model fit was unsatisfactory. However, by deleting item 8, the model fit significantly improved and was acceptable on nearly all of the indices, except for an elevated RMSEA. Therefore, it was deemed prudent to delete item 8 to proceed with a model for subsequent analysis. 

Second, a comparison of the fit indices of two models was conducted, including a three-factor model and one-factor model (which contained all items belonging to PNT). The purpose of this was to test whether CPNTT was consistent with the original PNTS study results. The results in Table 2 show that the one-factor model obviously did not fit the empirical data and no indices met the standard criteria. Thus, it can be seen that the three-factor structure was appropriate for the CPNTT.

Next, the CR and AVE of the three constructs were calculated based on the standardized factor loadings from Table 3. Note that the description of the items corresponds to the translated CPNTT, but is not a direct translation. The full CPNTT in Chinese can be found in Appendix A. The CR of autonomy thwarting was 0.81, with an AVE of 0.52; the CR of competence thwarting was 0.88, with an AVE of 0.72; while the CR of relatedness thwarting was 0.90, with an AVE of 0.69. Therefore, the CPNTT, as evaluated, was in accordance with the standards proposed by Fornell and Larcker [38]. Thus, convergent validity for the CPNTT was supported. As for discriminant validity, the correlation coefficient between autonomy thwarting and competence thwarting was 0.63, while the correlation coefficient between autonomy thwarting and relatedness thwarting was 0.41. The correlation coefficient between competence thwarting and relatedness thwarting was 0.23. As such, the squared values of these correlation coefficients were lower than the AVE of each construct, satisfying the requirement of discriminatory validity [38]. 

**Figure 1:**
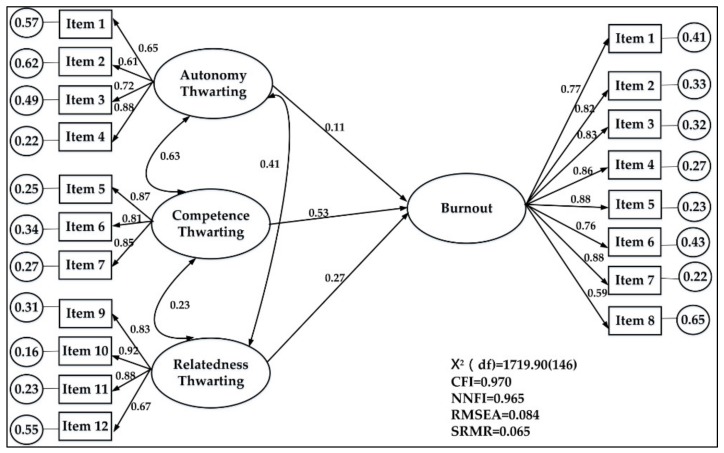
Structural equation model of the Chinese Psychological Need Thwarting of Teachers (CPNTT) on teacher burnout.

Using job burnout as a criterion variable, a structural equation model was utilized to examine the effects of PNT on teachers’ burnout. The results (Figure 1) illustrate that the model fits well with the empirical data and meets the standards for all indices. The three psychological need thwarting subscales can explain a significant amount of the variance in teacher’s burnout. The gamma coefficients from large to small are competence thwarting (0.53, *t* = 16.63, *p* < 0.01), relatedness thwarting (0.27, *t* = 11.49, *p* < 0.01), and autonomy thwarting (0.11, *t* = 3.60, *p* < 0.01). The *R^2^* of burnout is 0.54, indicating 54% of the variance in burnout is explained by the three PNT factors.

### 3.3. Test–Retest Reliability

**Figure 2:**
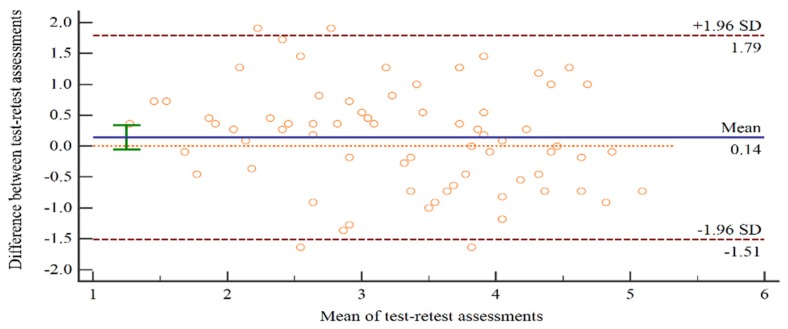
Bland–Altman plot for the differences between CPNTT measures from the two test sessions against the mean of the two test sessions for each subject. The 95% limits of agreement are indicated by the dashed lines.

After 6 months, the number of valid participants for the follow-up survey reduced to 74 due to transfers, resignations, or an unwillingness to complete the follow-up. In terms of test–retest reliability for the CPNTT, there was very little overall change in mean values between the two occasions (3.39 vs. 3.28, with *SDs* for both of 1.04). In terms of the indices used to evaluate the test–retest reliability, the results of paired *t* tests did not reach significance (*p* = 0.16), indicating no systematic bias from test occasion 1 to test occasion 2 (see Table 4). The ICC for the CPNTT was 0.90 and the SRD% was 27%, which met the criteria that the ICC value should be higher than 0.70 and the SRD% should be less than 30%. Therefore, the test–retest reliability of CPNTT is quite strongly supported. A Bland–Altman graph (See Figure 2) showed that, apart from four outliers, there was no heteroscedasticity observed. Moreover, the differences did not vary in any systematic way over the range of the CPNTT. However, it can also be noted that the SRD% of the three subscales of the CPNTT exceeded the standard of 30%, suggesting that the levels of random measurement error for the items were beyond the acceptable range when subscales were evaluated separately.

### 3.4. Measurement Invariance

After ensuring the validity of the factorial structures of the CPNTT, multiple group CFA was applied to detect whether teachers interpreted the scales differently according to gender (male and female), grade of instruction (primary or secondary school), or position (with or without concurrent administrative duties). The results (see Table 5) demonstrate that the CPNTT has support for scalar invariance across gender, grade of instruction, and position. Therefore, the result stated in Table 1 that male teachers had higher mean scores in terms of autonomy and competence thwarting as compared to female teachers can be attributed with higher confidence to differences in the construct, rather than different interpretations of the scale items. This is also the case in terms of the finding that that secondary teachers had higher mean scores for relatedness thwarting than primary school teachers.

Apart from grade of instruction, gender and position (whether or not concurrently serving as an administrative staff), all passed the invariance test with construct covariance constrained as equal. Therefore, only the test of partial measurement invariance was conducted for grade of instruction. However, even though the constraint was relaxed, ΔSRMR still violated the criterion for grade of instruction. In other words, the correlation between the three PNT subscales in the CPNTT were not equal for middle and primary school teachers.

## 4. Discussion

### 4.1. General Discussion

Due to an ongoing lack of measurement tools for evaluating PNT in China and considering the context-specific nature of PNT [20], this study translated the original version of the PNTS and systematically evaluated the reliability and validity of a Chinese version for teachers, the CPNTT. Based on the nature of teachers’ working environment in China’s basic education system, we evaluated a factor structure for the CPNTT that was generally consistent with other PNT studies (Martinent et al. [17], Liu et al. [18], and Cuevas et al. [28]), which included a three-factor model based on SDT, rather than a single-factor model (without differentiating autonomy thwarting, competence thwarting, and relatedness thwarting). Overall, the CPNTT developed in this study demonstrated good internal reliability, with acceptable cross-time consistency for the entire scale (i.e., test–retest consistency after 6 months). In terms of the evaluation of validity, the CPNTT has the support of several types of evidence. Specifically, concerning construct validity, the overall measurement model met the criteria of key indices, and both convergent and discriminant validities were supported through the calculation of CR and AVE. In addition, the three constructs of CPNTT were used to explain a significant proportion of the variance in teachers’ burnout (with an *R^2^* = 0.54), thereby supporting the scale’s criterion validity. It is worth mentioning that the CPNTT has characteristics of scalar invariance, and therefore, statistics comparing means (e.g., *t*-tests or *ANOVA*) can be used to examine differences in the CPNTT across groups without confounding the interpretation of items.

The results of the translation and evaluation of the validity of the CPNTT in this study concur with the suggestion by Bartholomew et al. [20] that the measurement of PNT must be designed in consideration of the characteristics of different contexts and in light of cultural or linguistic considerations. Through the process of this study, it was found that the characteristics of China’s educational culture influenced whether items could fit with the original, expected construct. Specifically, item 8 “Due to the lack of training opportunities in the environment, I feel that I am not competent in my daily work tasks” did not fit with the construct of competence thwarting for the CPNTT, as was expected, based on the original version of the PNTS. Although the latter part of the wording of this item apparently assesses thwarting of the teacher’s needs for competence (as in “I feel I am not competent in my daily work tasks”), the narrative context from the former part (namely, “Due to the lack of training opportunities in the environment”) caused confusion among participants and resulted in cross-factor loading with other factors, such as autonomy thwarting or relatedness thwarting. Since, in the environment of China schools, the opportunity for professional development is more strongly related to the influence of institutions and interpersonal relationships, it is understandable that cross-loading of factors occurred for this item. This situation is similar to previous studies which translated the PNTS and encountered situations where some items are inconsistent with the original Bartholomew et al. [20] scale, and deletion of items was required [17,18]. Thus, in this study, in order for the scale to better reflect the working environment of teachers in China’s basic education system, deleting this item was deemed appropriate to avoid degrading the quality of the overall measurement model. It should also be noted that the factor loading of item 8 in the Spanish teacher version of the PNTS was also the lowest [28], which might suggest the item has multiple interpretations or is influenced by other cross-cultural factors during translation. 

Based on the premise that the CPNTT is a reliable and valid tool for measuring PNT of teachers in China, a closer look at the results demonstrates that the most serious problem facing teachers is competence thwarting. Autonomy and relatedness thwarting are relatively less severe for Chinese teachers, as compared to competence thwarting, and are below the value of 4.45 indicating agreement, according to the criterion of Narli [37]. However, the degree of PNT in Chinese teachers is higher than the sample of Spanish physical education middle school teachers that were investigated by Cuevas et al. [28]. Given that this study and that of Cuevas et al. both used the teacher version of PNTS, such a comparison is meaningful, particularly in the absence of other international studies evaluating the PNT of teachers. Specifically, in the Cuevas et al. study, autonomy, competence, and relatedness thwarting were 1.76 (*SD* = 1.04), 2.00 (*SD* = 1.18), and 1.70 (*SD* = 0.94), respectively. The values of the corresponding variables in this study were 3.91 (*SD* = 1.61), 4.82 (*SD* = 1.81), and 2.09 (*SD* = 1.24). As far as we know, this is the first empirical study to directly investigate the PNT of teachers in China, since most SDT studies are only concerned with psychological need satisfaction, rather than thwarting [45]. 

Regarding the high psychological need thwarting of teachers in China, we speculate that one cause may be the teacher evaluation system based on student achievement performance implemented in the country. Too much emphasis on the performance of students, and resulting work pressure, are not entirely under the control of teachers, so competence thwarting is particularly high. This suggestion is consistent with the results of empirical research in the past, such as that of Feng [29], who suggested that Chinese teachers’ competence is relatively low and that there are few options for career development. Liu and Onwuegbuzie have also stated that job dissatisfaction factors specific to China have been identified as including high stress due to the national student assessment system and other teacher evaluation practices in China [46]. In addition, the teacher evaluation system based on student achievement performance may also be the reason why the relatedness thwarting of secondary school teachers is significantly higher than that of elementary school teachers in this study. This may be due to the fact that secondary schools pay more attention to students’ academic performance. A similar situation was found in Japan. In that context, teachers had less time for teacher collaboration and were more alienated due to pressures to pursue greater levels of individual performance, leading to less communication and collaboration and, presumably, a sense of relatedness thwarting [47,48].

A survey using the same measures of teacher burnout, by Yao et al. in China, found a mean value for emotional exhaustion of 3.10 (*SD* = 1.55) among school teachers in 2015 [49]. However, from the findings of this study, a mean value for emotional exhaustion of 4.06 (*SD =* 1.72) was found. This suggests that, quite possibly, over the past few years, the situation in China has become even more severe in terms of the expectations placed upon teachers, without the support and training required to deal with these expectations. These contextual factors could partially explain the relatively high levels of competence thwarting observed in this study, with a mean of 4.82 (*SD* = 1.81). Compared with other countries, the profession of teaching has a greater appeal to Chinese people, given the traditional emphasis on teacher education and teaching careers as being stable [46]. This feature may, to a certain degree, cushion the negative impact of school environments’ frustration of teachers’ psychological needs. However, given the relatively high level of PNT for all three subscales, as compared to the data from Spain, school administrators should not allow the existing situation of high levels of competence thwarting and moderate levels of autonomy and relatedness thwarting of teachers’ psychological needs to further worsen. This is particularly relevant given the strong relationship between PNT and teachers’ burnout evidenced by the results of this study, which clearly shows a positive and significant relationship between teachers’ PNT and eventual job burnout.

### 4.2. Practical Implications

SDT research has shown that the predictive power of PNT in terms of negative outcomes, such as burnout or lower levels of motivation, is stronger than for PNS [17,19]. As discussed above, it is a myth to assume that PNT and PNS exist on the same spectrum. As such, it seems clear that an emphasis on ameliorating or reducing the existence of thwarting in regards to teachers’ autonomy, relatedness, but particularly for competency (especially in the Chinese context) would be effective in both improving teaching and learning outcomes as well as reducing the emotional exhaustion that leads to teacher burnout and turnover. For teachers, thwarting of psychological needs typically leads to both burnout and an intention to leave the teaching profession. Teacher turnover not only is a waste of the effort and money by both teacher trainers and teachers in developing quality educators but also it results in the loss of qualified teachers in a country that needs greater, rather than fewer, teachers. In the environment of increasing teacher turnover within China’s basic education [50], the CPNTT developed by this study can be used as a practical tool for school administrators to directly investigate and evaluate the psychological needs of teachers in their school, while also conducting self-assessment in order to determine where and how teachers’ psychological needs may be thwarted. From the perspective of prevention, it may be too late to intervene when teachers have already grown tired of their profession. A more positive and proactive approach requires understanding from the source, classroom teachers, about how schools have thwarted teachers’ basic psychological needs. This will help to alert school administrators to the seriousness of teachers’ psychological needs and, in conjunction with efforts to satisfy teachers’ psychological needs, can further identify structural or systemic issues, which are simultaneously thwarting teachers’ self-determination. Furthermore, in addition to the social and contextual factors thwarting teachers’ psychological needs, individual-level efforts can be encouraged in order to prevent exhaustion and burnout. That is to say, for certain individuals, making social and contextual changes may not eliminate the personal or subjective sense of need thwarting and that individual efforts, in the absence of systemic change, can help in mitigating the damage of PNT. One such factor, that of resilience [51], has the potential to assist teachers in ameliorating or alleviating psychological need thwarting. As such, the use of the CPNTT as a means of teacher self-assessment and evaluation has further potential to provide teachers with the insights necessary to take proactive measures to engage in activities such as participation in teacher learning communities (to develop autonomy), self-study and professional development (to improve competence), and networking (to build relatedness).

### 4.3. Limitations and Future Research Directions

Despite the fact that the CPNTT demonstrates strong reliability and validity and provides unique empirical contributions and practical implications, the present study has certain limitations that should be addressed. First, although the test–retest reliability of the CPNTT is acceptable after 6 months, the SRD% for subscales, when measured individually, are slightly higher and do not meet the standard. Due to the limitation of a relatively large loss of data, due to transfers and retirements as well as participants’ unwillingness to complete the post-test, a broader evaluation of the test–retest consistency of the CPNTT should be conducted. Therefore, it is suggested that the CPNTT can be more specifically verified in the future in order to confirm its longitudinal measurement invariances, even over a time of more than 6 months.

Second, in the discussion part of this study, the majority of comparisons have been made with the data from Cuevas et al. [28]. Although some insights and perspectives can be obtained from international comparisons, such comparison must be done cautiously, particularly when it has not been confirmed whether or not both the CPNTT and Spanish physical education teacher’s PNTS have scalar invariance. Therefore, the future applications of the CPNTT, in terms of international or cross-cultural comparisons of measurement invariance, is a direction worthy of investigation. In addition, the relationships among coefficients between PNT and teacher burnout in this study were higher than those of Cuevas et al. [28]. As such, further research can use the CPNTT and related scales in other languages to evaluate whether or not the interpretations of teachers vary culturally. As was stated in the Introduction, differences exist between Western and Eastern cultures, more evidently in terms of autonomy, but possibly also in terms of competence and relatedness [29,30,31]. As such, this is a topic that can be evaluated by future research.

The presence of other moderating variables, such as the country, education system, or subject, on the relationship between teachers’ psychological needs thwarting and job burnout should be further explored in the future. Furthermore, the role of personal resilience in mitigating the effects of psychological need thwarting on teachers’ physical and mental condition, particularly in the context of teaching effectiveness and the prevention of teacher burnout, is a factor worthy of further study [51]. Additionally, how administrators can address improvements to the work environment to eliminate or reduce the thwarting of teachers’ psychological needs should be confirmed by continued research in this field. 

Finally, caution should be used when generalizing the results of the CPNTT, in terms of the covariance between constructs of the CPNTT being equal, for primary and secondary school teachers. These results must be interpreted cautiously, since the sample size of the two groups was very different (i.e., 1095 primary school teachers vs. 440 secondary school teacher). Naturally, larger samples of secondary school teachers would be useful for future evaluation of the CPNTT. Lastly, data on the overall distribution of teachers in China by gender, grade of instruction, years of experience, and position should be evaluated in order to determine the representativeness of the samples used for both test–retest reliability and confirmatory factor analysis. Further evaluation of differences based on gender and grade of instruction could also be conducted, since significant differences were found for males (in terms of higher levels of autonomy and competence thwarting) as compared to females, and higher levels of relatedness thwarting for secondary school teachers as compared to primary school teachers, although these differences had very small effect sizes and overall measurement invariance was found across these groups.

## 5. Conclusions

The CPNTT scale developed in this study has superb reliability and validity, as evidenced by strong goodness of fit, reliability, and validity. The scale has the potential to not only be used as a measure of the thwarting of teachers’ psychological needs in the Chinese context but also as a means for providing a basis for international comparative research, comparing both teachers’ psychological well-being and ill-being in different countries and contexts. The finding illustrate that the thwarting of Chinese teachers’ psychological needs is consistent with SDT theory and can be clearly defined by three subscales which, together, can explain a great deal of the variance in burnout among Chinese teachers (i.e., over 50% of the variance). Moreover, teachers in China’s basic education system have a high degree of frustration in terms of their psychological need for competence. This finding is presumed to be influenced strongly by the stringent teacher assessment system implemented in China. The resulting stress can influence not only teachers’ psychological well-being but also that of their students, making psychological need thwarting an issue of importance universally. If these forms of frustration are not handled properly, not only will classroom environments be negatively affected but continued thwarting of psychological needs will lead to greater degrees of burnout in the teaching profession, resulting in irreparable impacts on society.

## Figures and Tables

**Table 1:** Descriptive statistics, internal reliability, and between-subjects ANOVA.

Variables	*M*	*SD*	α	Background	*F*	Effect Size ^a^
Autonomy thwarting	3.91	1.61	0.76	Gender	59.10 *	0.04
				Grade of instruction	1.50	0.003
				Position	1.37	0.001
				Teaching subject	1.79	0.01
Competence thwarting	4.24	1.54	0.76	Gender	5.59 *	0.004
				Grade of instruction	1.26	0.002
				Position	0.41	0.00
				Teaching subject	1.66	0.01
Competence thwarting (removing item 8)	4.82	1.81	0.83	Gender	5.26 *	0.003
				Grade of instruction	0.76	0.001
				Position	0.56	0.00
				Teaching subject	1.86	0.01
Relatedness thwarting	2.09	1.24	0.81	Gender	1.71	0.001
				Grade of instruction	9.81 *	0.02
				Position	1.56	0.001
				Teaching subject	1.26	0.007
Emotional exhaustion	4.06	1.72	0.91	Gender	5.69 *	0.004
				Grade of instruction	4.48 *	0.003
				Position	1.97	0.001
				Teaching subject	1.81	0.011

Notes: ^a^ Partial eta squared was used for effect size; * *p* < 0.05.

**Table 2:** Indices of model fit for the CPNTT with different settings to evaluate item 8.

Setting	*Χ^2^*(*df*)	*CFI*	*NNFI*	*RMSEA*	*SRMR*
CPNTT (with item 8)	1374.386 (51)	0.921	0.898	0.130	0.134
CPNTT (without item 8)—3 factors	583.215 (41)	0.962	0.949	0.093	0.071
CPNTT (without item 8)—1 factor	6726.776 (44)	0.664	0.580	0.315	0.177

**Table 3:** Item properties of the CPNTT, including mean, standard deviation, item-total correlation, and loading.

Subscale (with Items)	*M* (*SD*)	Item-Total Correlation	Loading
**Autonomy Thwarting**			
Item1. Decision-making	3.07 (2.26)	0.63	0.65
Item2. Behaviors	4.69 (2.12)	0.61	0.61
Item3. Teaching methods	4.08 (2.17)	0.60	0.72
Item4. Conformity	3.83 (2.12)	0.73	0.88
**Competence Thwarting**			
Item5. Incapability	4.79 (2.13)	0.70	0.87
Item6. Situational incompetence	4.78 (2.06)	0.64	0.82
Item7. Awkwardness	4.90 (2.07)	0.67	0.85
**Relatedness Thwarting**			
Item9. Rejection	2.24 (1.68)	0.58	0.83
Item10. Indifference	1.95 (1.47)	0.54	0.92
Item11. Envy	2.39 (1.78)	0.51	0.88
Item12. Dislike	1.77 (1.28)	0.48	0.67

**Table 4:** Results for paired *t* tests, ICC, SEM, SRD, and SRD%.

Scale and Sub-scales	Pilot test	Follow-up	*p* ^a^	*ICC*	*SEM*	*SRD*	*SRD%*
CPNTT	3.39 (1.04)	3.28 (1.04)	0.16	0.90	0.33	0.91	0.27
Autonomy thwarting	3.95 (1.31)	3.73 (1.37)	0.22	0.83	0.55	1.52	0.39
Competence thwarting	4.50 (1.68)	4.25 (1.61)	0.07	0.87	0.59	1.64	0.37
Relatedness thwarting	1.99 (1.15)	2.10 (1.14)	0.56	0.90	0.36	1.00	0.49

Note: ^a^ *p* was based on paired *t*-tests.

**Table 5:** Measurement invariance across gender, grade of instruction and position.

	Configural Model ^a^	Loadings Constrained as Equal ^a^	Loadings and Thresholds Constrained as Equal ^a^	Loadings, Thresholds, and Covariance Constructs Constrained as Equal ^a^
**Gender**				
*Χ^2^*(*df*) or *ΔΧ^2^*(*Δdf*)	660.730 (82)	80.935 (8)	0 (8)	67.203 (3)
*CFI* or Δ*CFI*	0.959	−0.005	0.001	−0.004
*RMSEA* or *ΔRMSEA*	0.096	0.001	−0.004	0.003
*SRMR* or *ΔSRMR*	0.073	0.008	0	0.009
**Grade of instruction ^b^**				
*Χ^2^*(*df*) or *ΔΧ^2^*(*Δdf*)	658.335 (82)	68.452 (8)	0 (8)	82.648 (3)
*CFI* or Δ*CFI*	0.957	−0.004	0.001	−0.006
*RMSEA* or *ΔRMSEA*	0.097	0.0003	−0.005	0.004
*SRMR* or *ΔSRMR*	0.075	0.010	0	0.139
**Position**				
*Χ^2^*(*df*) or *ΔΧ^2^*(*Δdf*)	683.774 (82)	27.108 (8)	0 (8)	0.953 (3)
*CFI* or *ΔCFI*	0.957	0	0	0
*RMSEA* or *ΔRMSEA*	0.097	−0.002	−0.005	−0.002
*SRMR* or *ΔSRMR*	0.069	0.017	0	0.004

Note: ^a^ Configural models were reported using *χ^2^* (d*f*), *CFI*, *RMSEA*, and *SRMR*; other models were reported using *Δχ^2^* (*Δdf*), *ΔCFI*, *ΔRMSEA*, and *ΔSRMR*; ^b^ For convenience of comparison, junior and senior high school teachers were combined into one group (i.e., secondary school teachers) and then compared in terms of measurement invariance with primary teachers.

## Appendix A

**Items on the Chinese Version of a Psychological Need Thwarting Scale for Teachers**

Item 1. 我无法自己决定想要的教学方式

Item 2. 有在日常工作时,有股压力会影响我的行为举止,使其符合特定规范

Item 3. 我必需遵循某种规定的教学方式

Item 4. 我感受到外在环境的压力,限制我必须选定特定的教学方式

Item 5. 日常工作环境中有些情况让我觉得无能为力

Item 6. 有时我会跟人提到一些让我感到无能为力的事情

Item 7. 日常工作中,有些情况让我会产生无力感

Item 8.由于环境中缺乏磨练机会,我觉得自己不能胜任日常工作任务

Item 9. 我觉得自己与工作环境周围的人有所隔阂

Item 10. 我觉工作环境中的人对我冷漠

Item 11. 当我取得成功时,我觉得周遭的人会嫉妒我

Item 12. 我觉得学校中的人不喜欢我
